# Supplementary material for: HNF1A is a novel oncogene that regulates human pancreatic cancer stem cell properties
Source: eLife. 2018 Aug 3;7:e33947. doi: 10.7554/eLife.33947 (PMC6122955; doi:10.7554/eLife.33947)
Supplement: Supplementary file 1. — Limiting dilution assay was performed with sorted NY15 cells injected subcutaneously in NOD/SCID mice. The resultant numbers of tumors/injection is tabulated with estimated cancer stem cell frequencies calculated by extreme limiting dilution analysis (ELDA). [file elife-33947-supp1.docx]

**SUPPLEMENTARY FILE 1**

| Sub-  population (NY15) | 2000 cells/ animal | 200 cells/ animal | 20 cells/ animal | Estimated CSC frequency  (lower-upper confidence intervals) | Chisq  (vs. HH) | DF | Pr(>Chisq)  (vs. HH) |
| --- | --- | --- | --- | --- | --- | --- | --- |
| P1 | **1/5** | **0/5** | **0/5** | **1 in 10067**  **(71034-1427)** | **12.1** | **1** | **0.000492** |
| P2 | **5/5** | **2/5** | **0/5** | **1 in 406 (1250-406)** | **N/A** | **N/A** | **N/A** |
| P3 | **3/5** | **1/5** | **0/5** | **1 in 1869 (5288-1869)** | **4.12** | **1** | **0.0424** |

**Supplementary File 1: Cancer stem cell frequencies in PDA subpopulations** Limiting dilution assay was performed with sorted NY15 cells injected subcutaneously in NOD/SCID mice. The resultant numbers of tumors/injection is tabulated with estimated cancer stem cell frequencies calculated by extreme limiting dilution analysis (ELDA).
